# Side-chain Packing Using SE(3)-Transformer

**Published:** 2022

**Authors:** Akhil Jindal, Sergei Kotelnikov, Dzmitry Padhorny, Dima Kozakov, Yimin Zhu, Rezaul Chowdhury, Sandor Vajda

**Affiliations:** Department of Applied Mathematics and Statistics, Stony Brook University, Stony Brook, NY 11794; Department of Computer Science, Stony Brook University, Stony Brook, NY 11794; Department of Biomedical Engineering, Boston University, Boston, MA, 02215

**Keywords:** Protein Structure Prediction, Sidechain Packing, Deep Learning, Transformers

## Abstract

Predicting protein side-chains is important for both protein structure prediction and protein design. Modeling approaches to predict side-chains such as SCWRL4 have become one of the most widely used tools of its type due to fast and highly accurate predictions. Motivated by the recent success of AlphaFold2 in CASP14, our group adapted a 3D equivariant neural network architecture to predict protein side-chain conformations, specifically within a protein-protein interface, a problem that has not been fully addressed by AlphaFold2.

## 1. Introduction

The protein side-chain packing problem is important for both protein structure prediction (Kaden, Koch, and Selbig 1990) and protein design (Dahiyat and Mayo 1997). State-of-the-art approaches for predicting protein side-chain conformations, such as SCWRL4 (Krivov, Shapovalov, and Dunbrack 2009), deliver fast and highly accurate predictions making it one of the most widely used tools of its type. The interest to explore machine learning applications to predict side-chain orientations has grown over the last several years, as seen by recent works by Yanover et al. and Nagata et al. (Yanover, Schueler-Furman, and Weiss 2008; Nagata, Randall, and Baldi 2012). Motivated by the recent success of AlphaFold2 (Jumper et al. 2021) in CASP14, we adapted the SE(3)-Transformer neural network architecture (Fuchs et al. 2020) to predict protein side-chain conformations, specifically within a protein-protein interface, a problem that has not been fully addressed by AlphaFold2.

The SE(3)-Transformer architecture operates on 3D point clouds, takes advantage of the powerful self-attention mechanism, and adheres to equivariance constraints. These constraints ensure that network predictions are equivariant with respect to the global roto-translational transformations of the input point cloud, thus improving the robustness and overall performance. In our case, the point cloud represents the CA atoms of the protein, and we train the neural network to predict the key atom positions of the residue side chains given the positions of the neighboring backbone atoms.

## 2. Methods

**Figure 1.**
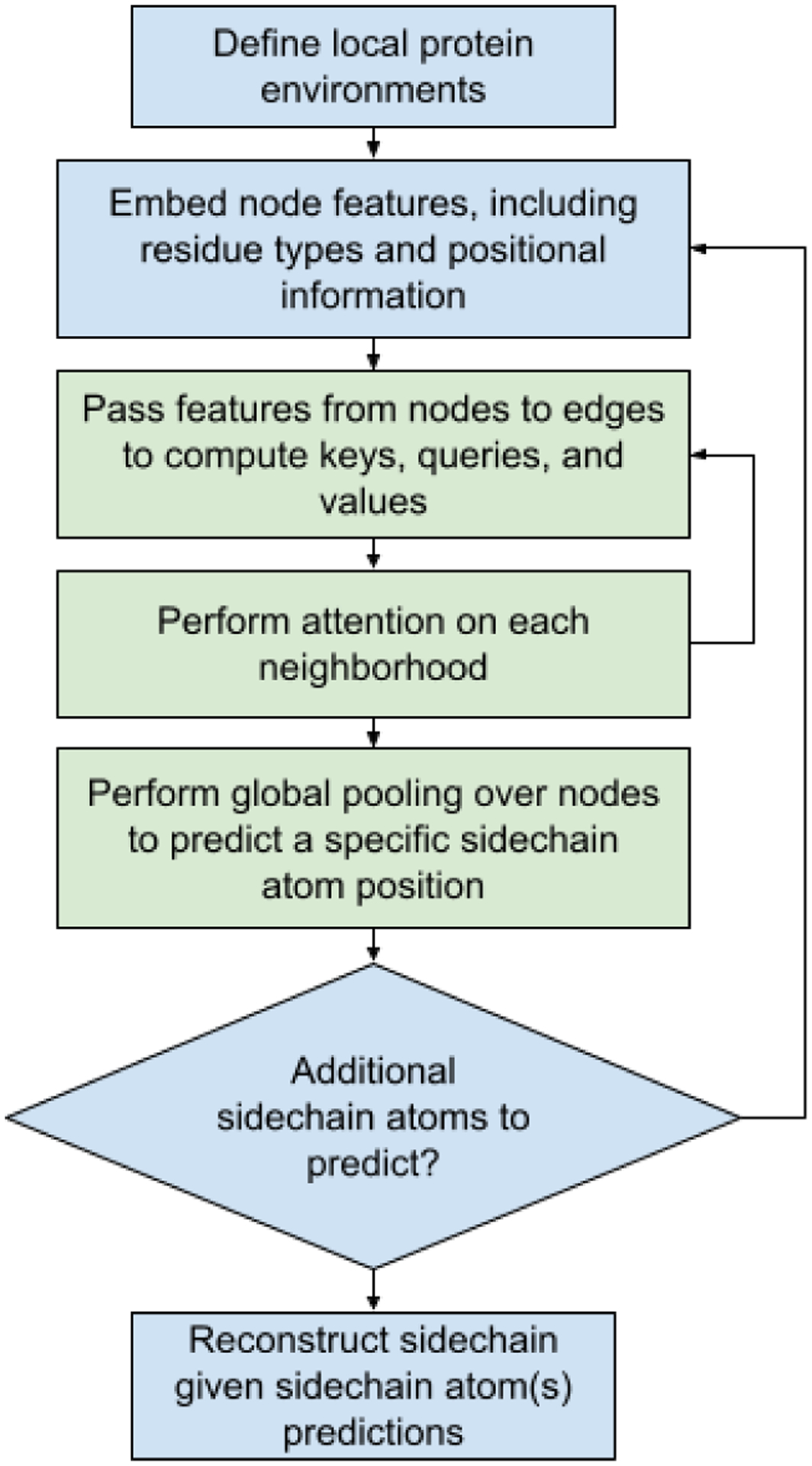
Overall Process Flow for Sidechain Prediction. Green boxes indicate the portions of the process which are within the SE(3)-Transformer architecture. Blue boxes indicate upstream and downstream processes for the SE(3)-Transformer.

The side-chain prediction method explored in this work is schematically represented in Fig. 1 and consists of the following steps. First, for each residue, we define a local environment composed of its spatial neighbors. Second, these neighboring residues are treated as graph nodes, and the corresponding node features embed positional and residue-type information. The resulting graphs serve as inputs to the SE(3)-Transformer and are processed by several consecutive SE(3)-equivariant attention layers. Subsequently, a global pooling over the nodes is used to predict a designated sidechain atom position (either the χ^−2^ distal atom or the functional end-group atom) of the central residue. Finally, a full atomic representation of the side-chain orientation is constructed by identifying a rotamer from a library having a sidechain atom that is nearest to the prediction(s).

### 2.1. Neighborhood Graph Representation

A protein containing *N* residues is represented as a collection of neighborhoods, *N*_*i*_ ∈ {1, …, *N*}, where each neighborhood *N*_*i*_ is centered on a residue *i* and is defined based on plausible interactions between residue *i* and any residue *j* within a local environment. Specifically, a pair of residues, *i* and *j*, are deemed to have a plausible interaction if any pair of backbone-dependent rotamers of *i* and *j* result in an interatomic distance of less than 5Å. In the context of the attention mechanism described below, considering local neighborhoods allows reducing the computational complexity from quadratic to linear in the number of residues.

### 2.2. The SE(3)-Transformer Architecture

In this work, we rely on the SE(3)-Transformer architecture by Fuchs et al. 2020. Below, we give a brief overview of a single layer of the SE(3)-Transformer.

In a standard self-*attention mechanism* (Vaswani et al. 2017), three vectors are considered for each token: query vector *q*_*i*_ ∈ *R*^*p*^, key vector *k*_*i*_ ∈ *R*^*p*^ and value vector *v*_*i*_ ∈ *R*^*r*^ for *i* = 1, …, *n*, where low dimensional embeddings have dimensions *r* and *p*. These vectors are the outputs of learnable functions of token feature vectors *f*_*i*_ ∈ *R*^*d*^:

(1) $$q_{i}=h_{q}\left(f_{i}\right),\;k=h_{K}\left(f_{i}\right),\;v=h_{V}\left(f_{i}\right)$$

Based on these vectors, we can calculate the attention weights and attention-weighted value messages:

(2) $$\text{Attn}\left(q_{i},\left\{k_{j}\right\},\left\{v_{j}\right\}\right)=\sum_{j=1}^{n}\alpha_{ij}v_{j}\;\;\alpha_{ij}=\frac{exp\left(q_{i}^{T}k_{j}\right)}{\sum_{j^{\prime }=1}^{n}exp\left(q_{i}^{T}k_{j^{\prime }}\right)}$$

When applied to 3D point cloud data, each token *i* is associated with a geometric coordinate *x*_*i*_ ∈ *R*^3^.

In many practical applications, the outputs of the function being learned do not change or change accordingly with translational and rotational transformations of the inputs, that is, the function possesses the properties of invariance or equivariance to the SE(3) group of roto-translational transformations. These two properties represent important symmetries of a problem, and while a general neural network can learn to respect these symmetries, explicitly incorporating symmetry constraints into the neural network can be more efficient with respect to the number of learnable parameters and amount of data required for training. One successful example of such symmetryaware architecture is the Tensor Field Network (Thomas et al., 2018), which maps point clouds to point clouds in 3D while respecting the SE(3)-equivariance.

Recently, an expansion of this architecture incorporating the attention mechanism was implemented in the SE(3)-Transformer. In this architecture, the input is a feature vector field *f* : *R*^3^ → *R*^*d*^ defined on a discrete set of points in space - point cloud:

(3) $$f(x)=\sum_{j=1}f_{j}\delta \left(x-x_{j}\right),$$

where δ is the Dirac delta function, {*x*_*j*_} are the 3D point coordinates, and *f*_*j*_ ∈ *R*^*d*^ is a concatenation of vectors $f_{j}^{l}\in R^{2l+1}$ of different degrees *l* of SO(3)-group irreducible representations: $f_{j}={⊕}_{l\geq 0}f_{j}^{l}$.

A learnable attention-based transformation of this vector field satisfying the SE(3) equivariance can be expressed as:

(4) $$f_{out,i}^{l}=w_{V}^{ll}f_{in,i}^{l}+\sum_{k\geq 0}\sum_{j\in Ni\backslash i}\alpha_{ij}\;W_{V}^{lk}\left(x_{j}-x_{i}\right)f_{in,j}^{k}$$

Here $w_{v}^{ll}$ is a learnable scalar, *a*_*ij*_ is a scalar attention weight, and $W_{V}^{lk}\left(x_{j}-x_{i}\right):R^{3}\to R^{\left(2k+1)(2l+1\right)}$ is a learnable weight kernel from degree *k* to degree *l*, which can be written as:

(5) $$W^{lk}(x)=\sum_{J=|k-l|}^{k+l}{\varphi_{J}}^{lk}(|x|){W_{J}}^{lk}\left(\frac{x}{\left|x\right|}\right),\;{W_{J}}^{lk}\left(\frac{x}{\left|x\right|}\right)=\sum_{m=-J}^{J}Y_{Jm}\left(\frac{x}{\left|x\right|}\right){Q_{Jm}}^{lk}$$

Where ${\varphi_{J}}^{lk}(|x|):R^{3}\to R$ is a learnable radial neural network, is ${W_{J}}^{lk}\left(\frac{x}{\left|x\right|}\right):R^{3}\to R^{\left(2k+1)(2l+1\right)}$ a non-learnable angular kernel from degree *k* to degree *l, $Y_{Jm}\left(\frac{x}{\left|x\right|}\right):R^{3}\to R$* is a spherical harmonic, and $Q_{Jm}^{lk}\in R^{\left(2k+1)(2l+1\right)}$ are Clebsch-Gordon coefficients.

The equivariance of the transformation is ensured by the fact that the learnable weight kernel *W*^*lk*^ (*x*) is expressed as a linear combination of the non-learnable angular kernels ${W_{J}}^{lk}\left(\frac{x}{\left|x\right|}\right)$ with the scalar radial function ${\varphi_{J}}^{lk}(|x|)$ coefficients and thus performs a valid conversion of degree *k* vector to degree *l* vector.

Furthermore, invariant attention weights are achieved via a dot-product attention structure, as shown below:

(6) $$\alpha_{ij}=\frac{exp\left(q_{i}^{T}k_{ij}\right)}{\sum_{j^{\prime }\in N_{i}\backslash i}exp\left(q_{i}^{T}k_{ij^{\prime }}\right)},\;q_{i}={⊕}_{l\geq 0}\sum_{k\geq 0}W_{Q}^{lk}f_{in,i}^{k},\;k_{ij}={⊕}_{l\geq 0}\sum_{k\geq 0}W_{K}^{lk}\left(x_{j}-x_{i}\right){f^{k}}_{in,j}$$

Wherein this mechanism consists of a normalized inner product between a query vector q at node *i* and a set of key vectors ${\left\{k_{ij}\right\}}_{j\in N_{i}\backslash i}$ along each edge *ij* in the neighborhood *N*_*i*_ of node *i*. This architecture can be easily generalized by introducing several channels per representation degree.

### 2.3. Node Features

Our goal was to predict the positions of specific side-chain atoms given the positions of the backbone atoms of the neighboring residues, the so-called side chain packing problem In our implementation, each point of the 3D cloud represents a single residue, more specifically, the coordinate of the CA atom of that residue. We use two 3D vectors (i.e. degree *l*=1) input feature vectors to represent the relative positions of the C and N atoms of the same residue with respect to the CA atom. In addition, we use 20 scalar (degree *l*=0) input features to represent one-hot encoding of the residue type.

### 2.4. Final Layer

The final layer of the SE(3)-Transformer is set to produce one scalar (degree l=0) feature *s*_*i*_ and one 3D vector (degree *l*=1) feature e *v*_*i*_ per node. The final prediction *p* of the specific side chain atom position is made by performing a global pooling over the neighborhood nodes using scalar features as weights for the vector features: $p=\sum_{i}s_{i}v_{i}+x_{i}$ where *x*_*i*_ is the coordinate of the CA atom of the residue represented by node *i*.

### 2.5. Rotamer Selection

Upon predicting the χ^−2^ distal atom and the functional end-group atom, we reconstruct the side-chain conformation by selecting the rotamer with the lowest 2-atom RMSD from a library of backbone-dependent rotamers (PyMOL; (Krivov, Shapovalov, and Dunbrack 2009)).

### 2.6. Experiments

The deep-learning model was trained on a PISCES PDB (Wang and Dunbrack 2003) corpus, containing PDB structures with less than 2.0Å resolution, and clustered at an 80% sequence similarity. The corpus was modified to exclude any PDB entries which shared up to a 30% sequence similarity with the PDB entries used in the test dataset. In this experiment, the focus was to determine the accuracy of predicting side-chain conformations within a protein-protein interface. Accordingly, the test dataset was based on the ‘easy’ binary protein-protein complexes from Protein-Protein Docking Benchmark 5.5 (Vreven et al. 2015), totaling to 72 cases. ‘Easy’ protein-protein complexes were used to test the self-attention model, because the training of the model relied upon accurate backbone coordinate information, which may not be present in the ‘harder’ cases from the Protein-Protein Docking Benchmark 5.5.

The trained deep-learning model was tested based on four types of experiments for side-chain prediction: (1) unbound proteins at a protein-protein interface, (2) bound proteins at a protein-protein interface, (3) unbound protein at a protein-protein interface without its binding partner, and (4) bound protein at a protein-protein interface without its binding partner. For the unbound cases, (1) and (3), the unbound proteins were superimposed onto the respective bound cases. Furthermore, the trained model was used to predict at least one side-chain coordinate: (1) the distal χ^−2^ atom and (2) the functional end-group atom (Beglov et al. 2012).

Subsequent to predicting the above-identified atomic coordinates for each sidechain, the sidechain was reconstructed based on a backbone dependent rotamer library (Shapovalov and Dunbrack 2011). The rotamer was selected based on an RMSD minimization between the at least one predicted atomic coordinate, and the corresponding atomic coordinate of the discrete rotamer.

Finally, side-chain predictions were carried out for the same four experiments using SCWRL4, and are presented in the results section. We trained the SE(3)-Transformer with 7 layers having up to 4 representation degrees and a total of 32 channels. 20 epochs at an initial learning rate of 1e-3 and a batch size of 100 were used.

## 3. Results

**Figure 2:**
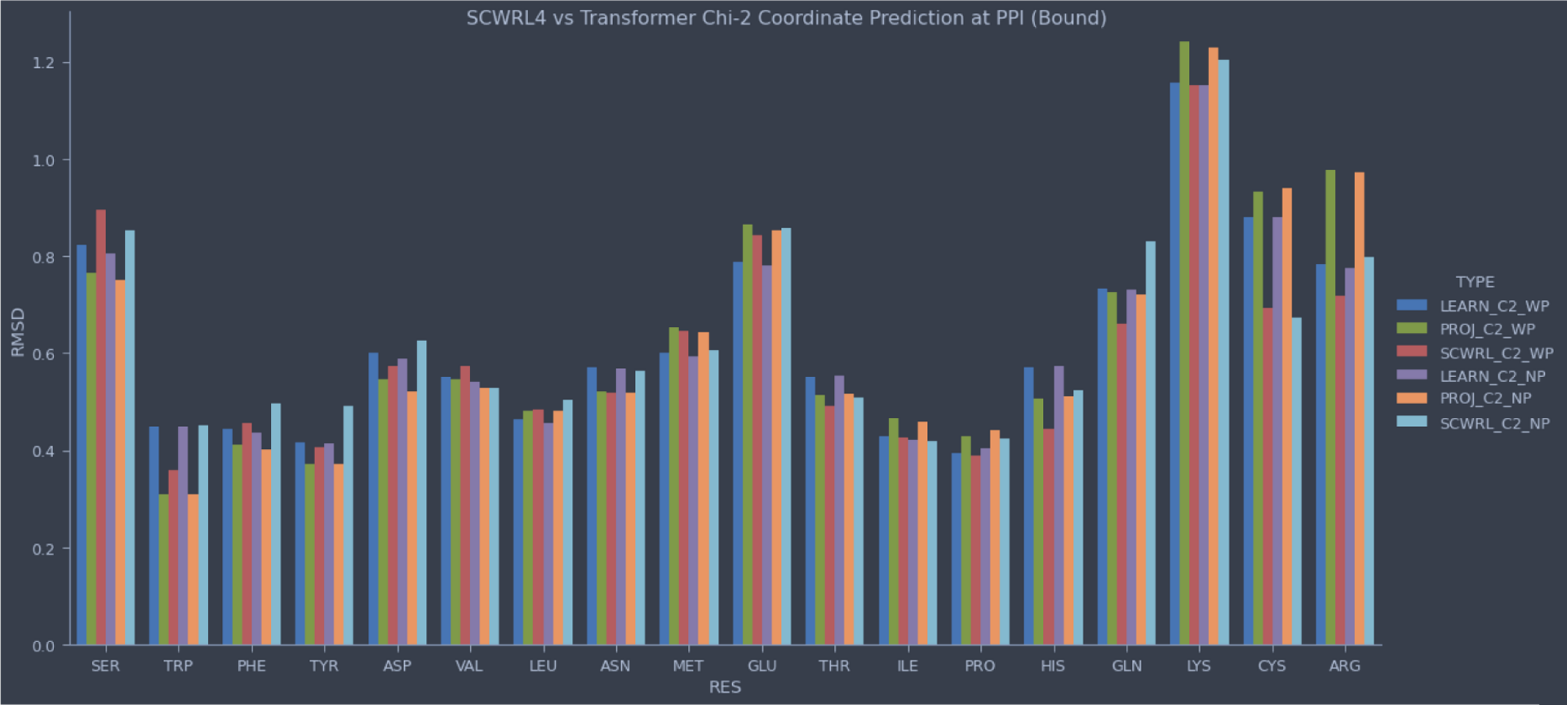
SCWRL4 vs SE(3)-Transformer Chi-2 Distal Coordinate Predictions at a Protein-Protein Interface for Bound Proteins. The RMSD for all predictions are presented. In blue is the SE(3) learning prediction for Chi-2 with its protein-partner. In green is the SE(3) learning prediction that is mapped to the closest SCWRL rotamer. In red is the SCWRL prediction with its protein-partner. In purple is the SE(3) learning prediction for Chi-2 without its protein-partner. In orange is the SE(3) learning prediction (without a partner) that is mapped to the closest SCWRL rotamer. In cyan is the SCWRL prediction without its protein-partner.

**Figure 3:**
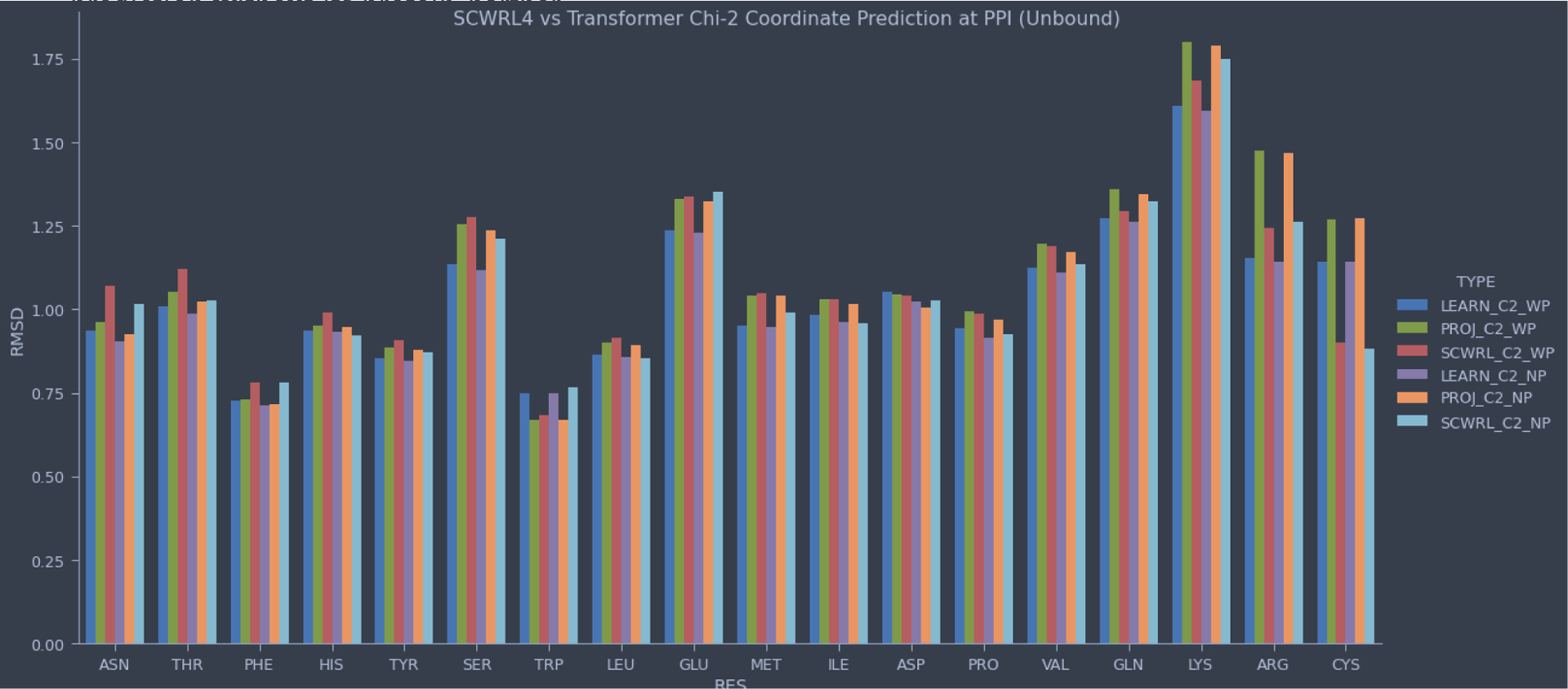
SCWRL4 vs SE(3)-Transformer Chi-2 Distal Coordinate Predictions at a Protein-Protein Interface for Unbound Proteins. The RMSD of all predictions are presented. In blue is the SE(3) learning prediction for Chi-2 with its protein-partner. In green is the SE(3) learning prediction that is mapped to the closest SCWRL rotamer. In red is the SCWRL prediction with its protein-partner. In purple is the SE(3) learning prediction for Chi-2 without its protein-partner. In orange is the SE(3) learning prediction (without a partner) that is mapped to the closest SCWRL rotamer. In cyan is the SCWRL prediction without its protein-partner.

**Figure 4:**
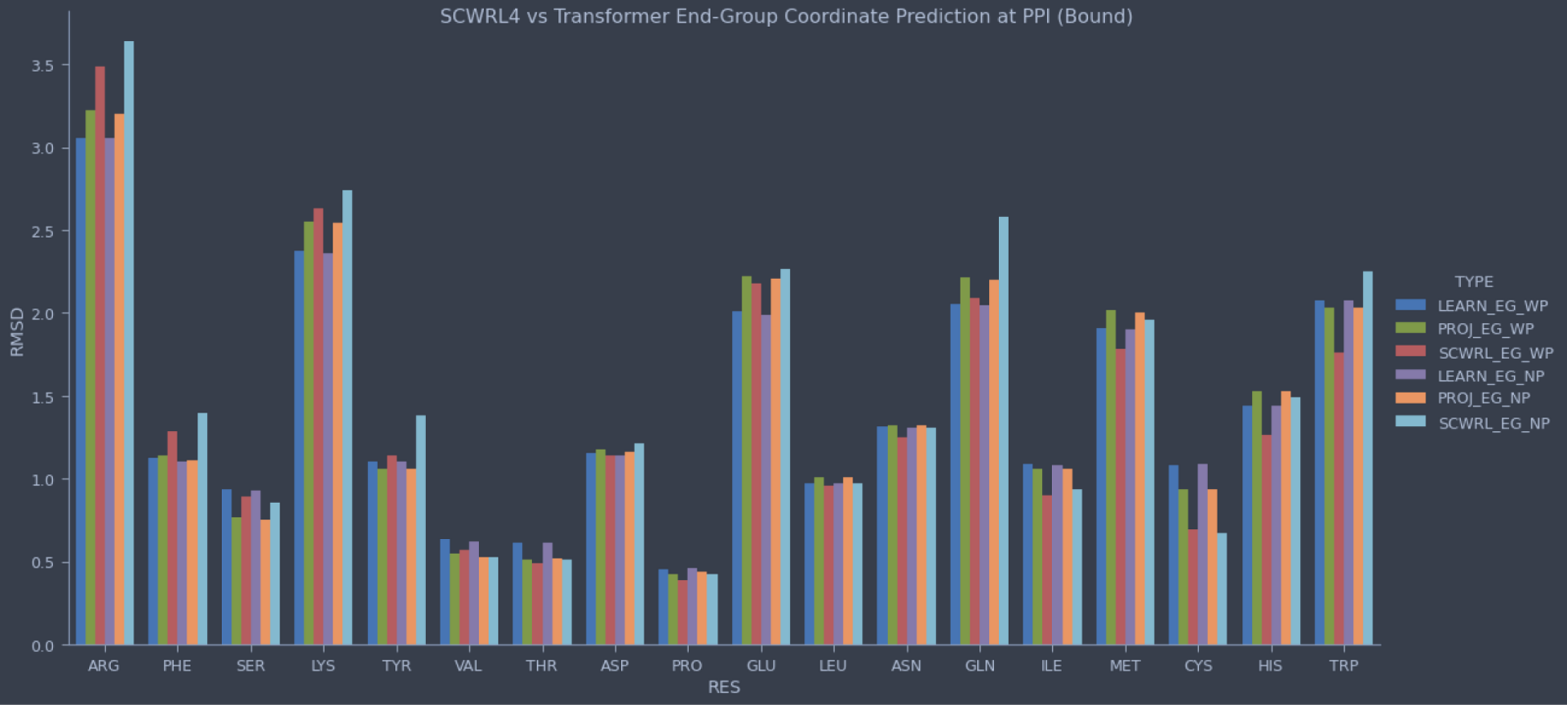
SCWRL4 vs SE(3)-Transformer Functional End-Group Coordinate Predictions at a Protein-Protein Interface for Bound Proteins. The RMSD of all predictions are presented. In blue is the SE(3) learning prediction for end-group with its protein-partner. In green is the SE(3) learning prediction that is mapped to the closest SCWRL rotamer. In red is the SCWRL prediction with its protein-partner. In purple is the SE(3) learning prediction for end-group without its protein-partner. In orange is the SE(3) learning prediction (without a partner) that is mapped to the closest SCWRL rotamer. In cyan is the SCWRL prediction without its protein-partner.

**Figure 5:**
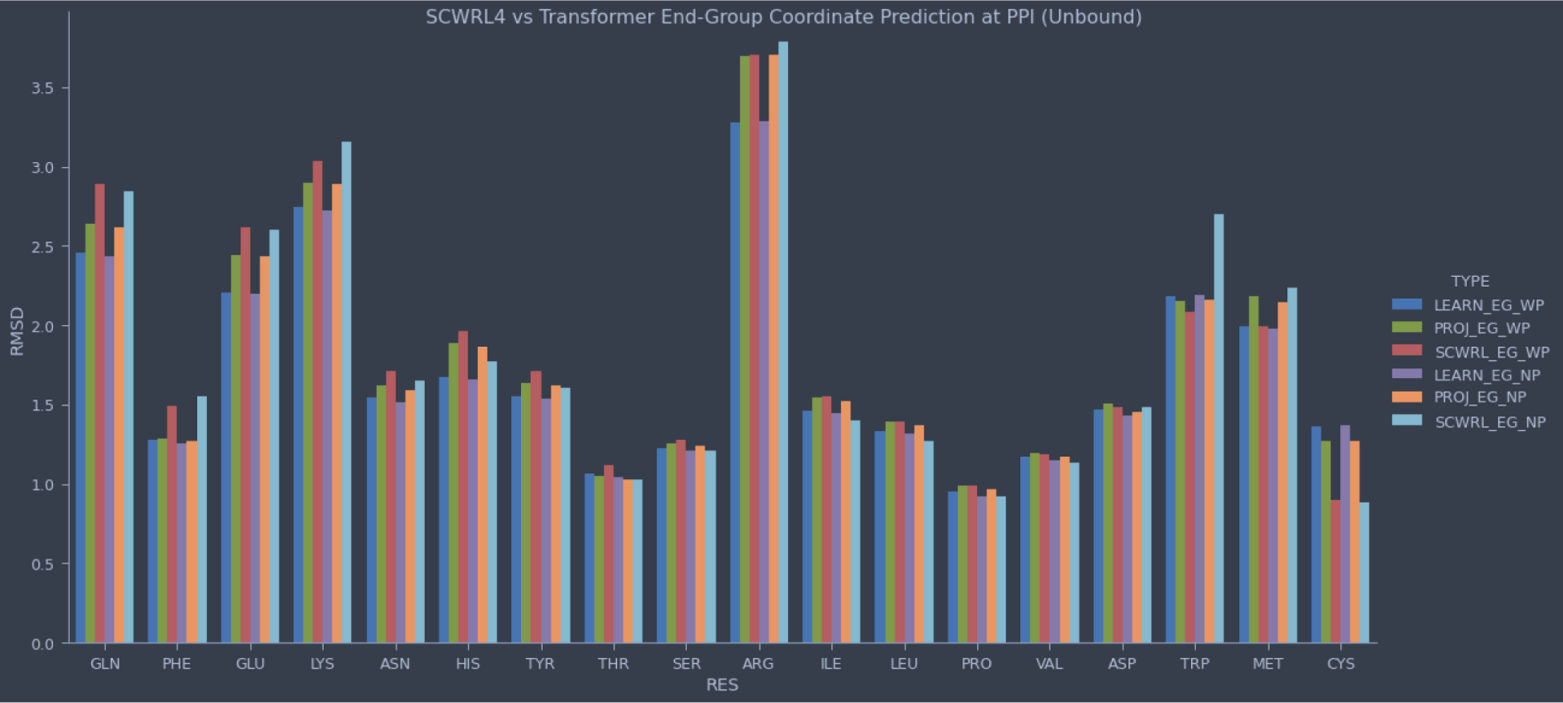
SCWRL4 vs SE(3)-Transformer Functional End-Group Coordinate Predictions at a Protein-Protein Interface for Unbound Proteins. The RMSD of all predictions are presented. In blue is the SE(3) learning prediction for end-group with its protein-partner. In green is the SE(3) learning prediction that is mapped to the closest SCWRL rotamer. In red is the SCWRL prediction with its protein-partner. In purple is the SE(3) learning prediction for end-group without its protein-partner. In orange is the SE(3) learning prediction (without a partner) that is mapped to the closest SCWRL rotamer. In cyan is the SCWRL prediction without its protein-partner.

The performance of the trained model on a test set and its comparison with the performance of SCWRL is presented in Fig. 2–5. The results of the distal χ−2 atom prediction on the unbound and bound datasets are shown in Fig. 2 and Fig. 3, respectively. Similarly, Fig. 4 and Fig. 5 represent the results for end-group coordinate prediction on the unbound and bound datasets. While, overall, we predict the side-chain conformations with an accuracy comparable to SCWRL4, the performance of the two methods is consistently different on the unbound and bound datasets, with SCWRL outperforming our approach on the bound dataset, and our approach providing better results on unbound structures.

In all figures, the notation ‘LEARN’ refers to the SE(3)-Transformer results, ‘SCWRL’ refers to the SCWRL4 prediction, ‘PROJ’ refers to the projection prediction based on the side-chain reconstruction, ‘C2’ refers to the distal χ^−2^ atom prediction, ‘EG’ refers to the functional end-group atom prediction, ‘WP’ refers to the prediction with the protein partner included in as part of the input, and ‘NP’ refers the prediction without the protein partner included as part of the input.

## 4. Conclusion

This manuscript describes our attempt to apply the SE(3)-equivariant transformer architecture to the problem of predicting the protein residue side chain orientations. The classical methods attempting to solve the same problem usually approach it by performing a combinatorial search over the side-chain orientation space and trying to find a minimal energy solution. Here, we demonstrate that a novel SE(3)-equivariant transformer architecture can be straightforwardly used to learn a solution to the same problem given enough training data. We test the approach on a task of reconstructing the side-chains in protein interfaces, using both bound and unbound subunit structures. The quality of the resulting predictions is comparable to that of the best-in-class classical methods. We suggest that this type of approach could be used as a part of larger network architectures dedicated to solving problems related to protein structure, in particular those used for prediction and design of protein complexes.
